# Guiding Clinical Prescription of Topical Extemporaneous Formulations of Sodium Cromoglycate Based on Pharmaceutical Performance

**DOI:** 10.3390/pharmaceutics15061609

**Published:** 2023-05-29

**Authors:** Olga González-González, Enrique Leal, Mercedes Martín-Martínez, Liliana Bautista, Maria Paloma Ballesteros, Juan J. Torrado, Dolores R. Serrano

**Affiliations:** 1 Departamento de Farmacia Galénica y Tecnología Alimentaria, Facultad de Farmacia, Universidad Complutense de Madrid, Plaza Ramón y Cajal, s/n, 28040 Madrid, Spain; 2 Community Pharmacy, 140 Canillas Road, 28043 Madrid, Spain; 3 Instituto de Química Medica, Juan de la Cierva, 28006 Madrid, Spain; 4 Instituto Universitario de Farmacia Industrial (IUFI), Facultad de Farmacia, Universidad Complutense de Madrid, Plaza Ramón y Cajal, s/n, 28040 Madrid, Spain

**Keywords:** cromoglycate, extemporaneous compounding formulations, stability, skin permeability

## Abstract

Cromoglycate (SCG) is widely used for allergy processes, and inflammatory states acting as a mast cell membrane stabilizer that inhibits the histamine and mediator release. Currently, SCG topical extemporaneous compounding formulations are prepared in hospitals and community pharmacies, as no industrial fabricated medicines are available in Spain. The stability of these formulations is unknown. Additionally, there are no clear guidelines on which concentration and vehicle are more suitable to enhance permeation across the skin. In this work, the stability of commonly prescribed topical SCG formulations in clinical practice was evaluated. Different vehicles commonly employed by pharmacists daily for formulating topical SCG were investigated (Eucerinum, Acofar Creamgel, and Beeler’s base) at different concentrations, ranging from 0.2 to 2%. The stability of topical extemporaneous compounded SCG formulations can be extended for up to three months at room temperature (25 °C). Creamgel 2% formulations significantly improved the topical permeation of SCG across the skin, being 4.5-fold higher than formulations prepared with Beeler’s base. The reason attributed to this performance can be related to the lower droplet size formed upon dilution in aqueous media combined with a lower viscosity, which facilitates its application and extensibility on the skin. The higher the SCG concentration in Creamgel formulations, the higher the permeability across both synthetic membranes and pig skin (*p*-value < 0.05). These preliminary results can be used as a guide to prompt a rational prescription of topical SCG formulations.

## 1. Introduction

**Figure 1:**
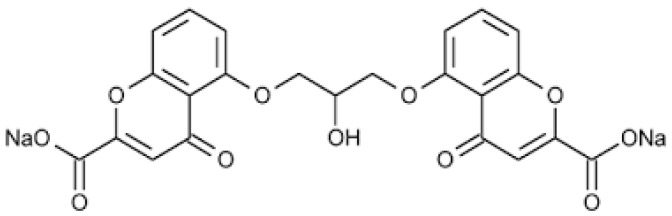
Chemical structure of SCG.

Cromoglycate sodium (cromolyn sodium, SCG) is an antioxidant and anti-inflammatory drug that belongs to the chromone group, a series of components with the chemical structure 5:6 benzo-1:4 pyrone, widely used for allergies processes and inflammatory states (Figure 1). SCG is classified as a mast cell membrane stabilizer that inhibits the histamines and mediators released through these cells [1].

Despite its unknown mechanism of action, it is capable of inhibiting the secretion of mast cell mediators through guanosine-5′-triphosphate and modulating sensory nerve fibers [2,3]. Histamine is released not only in physiological processes, but also in allergic states and immediate hypersensitivity reactions in a very explosive manner. This release is produced by multiple physical (hot, cold, radiations, etc.) and chemical agents (antigens, calcium, anaphylatoxins, cytotoxic agents, etc.), which produce chain reactions that increase intracellular calcium levels either by facilitating its extracellular penetration or promoting its mobilization from internal tanks [4]. In addition, cromoglycate has an action on B lymphocytes by decreasing the production of immunoglobulin E, and therefore inhibiting immediate hypersensitivity allergic reactions, and on neutrophils, inhibiting the production of the superoxide anion that causes tissue damage [5], differing from other mast cell stabilizer drugs [6].

Currently, this drug is used for prophylaxis of mild-to-moderate bronchial asthma, and adjunctive treatment of allergic rhinitis and systemic mast cell disease (mastocytosis, mast cell activation syndrome) in pediatric patients and adults [7,8]. It can be administered by different routes such as the inhalation route for asthma, as an ophthalmic solution for conjunctivitis, topically for dermatitis states and the prevention of itching, and orally for mast cell pathologies and food allergies [9]. Several medicines containing SCG are commercially available for pulmonary administration as dry powder inhalers, oral administration such as Gastrocrom^®^ in the USA, and eye drops (Opticrom^®^). To the best of our knowledge, no industrial medicines are commercialized for skin use, which makes the elaboration of extemporaneous compounding formulations for treating allergic pathologies necessary, especially in the pediatric population [10]. Moreover, oral, ophthalmic, and pulmonary medicines are not available in all countries, making their elaboration in hospitals or community pharmacies required. However, the industrial commercialization of topical formulations of SCG is gaining attention, as several clinical trials are currently ongoing. For example, a combination product for topical use containing SCG (5%), diphenhydramine (1%), and trolamine salicylate (10%) in an emollient cream base is being evaluated in a clinical trial for the treatment of cutaneous lesions of mastocytosis (https://beta.clinicaltrials.gov/study/NCT04846348, accessed on 20 April 2023). Additionally, a novel application for the topical use of SCG is under evaluation in clinical trials for the treatment of rosacea-associated erythema (https://clinicaltrials.gov/ct2/show/NCT01933464, accessed on 20 April 2023).

The SCG permeation across the skin is expected to be low, considering its physicochemical properties (MW 512 Da and log D between −3 and −5) [11]. Mast cells are localized in the dermis, near blood vessels, nerves, and hair follicles, under physiological conditions. However, mast cells can accumulate in chronic atopic dermatitis lesions and even migrate into the epidermis [12]. Based on this, topical formulations should ensure that the drug reaches mast cells to elicit a pharmacological effect in the outermost layer and interface of the epidermis (superior layer of the skin) called the stratum corneum [13,14].

Nowadays in Spain, there are only formulations marketed as eye drops (Cusicrom^®^). However, the REMA (Red Española de Mastocitosis) considers cromoglycate cream, at a concentration between 0.2% and 4.0%, the first-line treatment in pediatric mastocytosis and the oral administration of cromoglycate solutions in the case of diffuse cutaneous mastocytosis [9,15,16]. Currently, extemporaneous compounding formulations are prepared in community pharmacies with a limited expiry date (7–15 days) on a routine basis due to the lack of scientific information regarding the SCG stability profile. To reduce the lack of efficacy promoted by the degradation of the drug on prolonged treatments, patients go weekly to collect their formulations from community pharmacies. If the chemical stability of topical extemporaneous compounded formulations were estimated, formulations would be prepared and collected from the community pharmacies accordingly to their expiry date, enhancing patient compliance and promoting a better management of resources.

Currently, topical SCG formulations are commonly prescribed at 0.2% in Spanish pharmacies, with low shelf-life stability due to the clinical evidence for prolonged periods. Efficacy is highly variable and tends to be more limited in adults compared to children. Even though higher concentrations of topical extemporaneous compounded formulations are rarely prescribed in Spain, in other countries, clinical trials using much higher concentrations have shown promising results [15]. For this reason, the first objective of this work was to determine the physicochemical stability of a range of topical SCG formulations commonly prepared by the Spanish Community pharmacies for pediatric patients at the conventional concentration prescribed (0.2%). Secondly, topical SCG formulations prepared at higher concentrations than 0.2% will be compared in terms of their in vitro permeability across a synthetic Strat M-membrane^®^ and porcine ear skin, which is the closest in structure to the human skin to provide evidence of the effect of concentration and vehicle used on skin permeability, to guide clinical prescriptions based on the severity of the lesions being treated and the patient characteristics.

## 2. Materials and Methods

### 2.1. Materials

Cromoglycate sodium (SCG) was purchased from Fagron (Barcelona, Spain), batch number 244042 (purity > 99.9%). The aqueous bases (O/W), Creamgel, Beeler’s base, and Eucerinum^®^ for the preparation of the topical formulations were also purchased from Acofar (Barcelona, Spain). Beeler’s base consists of water, cetyl alcohol, propylene glycol, beeswax, sodium laureth sulfate, glyceryl stearate SE, phenethyl alcohol, phenoxyethanol, and caprylyl glycol. The Creamgel composition specified by the supplier is water (≥50%), butylene glycol (1–5%), liquid paraffin (1–5%), dimethicone (1–5%), cetyl alcohol (1–5%), polyacrylate 13 (1–5%), polyisobutylene (0.1–1%), alkyl acrylate crosspolymer (0.1–1%), diazolidinyl urea (0.1–1%), sodium benzoate (0.1–1%), polysorbate 20 (0.1–1%), potassium sorbate (0.1–1%), tetrasodium EDTA (≤0.1%), and BHT (≤0.1%). Eucerinum base contains isopropyl palmitate, glyceryl stearate, glycerin, PEG5 stearyl stearate, mineral oil, petrolatum, benzyl alcohol, dimethicone, lanolin alcohol, phenoxyethanol, steareth-21, sodium hydroxide and demineralized water. Acetonitrile (HPLC grade) was purchased from Scharlab (Madrid, Span), while the phosphate buffer (analytical reagent grade) was obtained from Panreac (Madrid, Spain). The purified water was obtained through an Elix 3 Millipore purified water system (Merck, MA, USA). All other chemicals were of analytical grade and used without further purification.

### 2.2. Methods

#### 2.2.1. Preparation of SCG Topical Formulations

Three topical O/W formulations were prepared in triplicate, ranging from 0.2 to 2% using different vehicles, Eucerinum^®^, Acofar Creamgel, and Beeler’s base. The formulations were packaged in a 5 mL propylene plastic container (Jose Mestre, Madrid, Spain). Each formulation was codified accordingly, as described in Table 1. The first set of stability experiments at 25 and 40 °C was performed with 0.2% SCG in Eucerinum^®^ and Acofar Creamgel base, without propylene glycol as the wetting agent. The second set of stability experiments was carried out with Acofar Creamgel and Beeler’s base at 1 and 2% SCG. The formulations were tested at 3 and 6 months, at the same conditions as in the first study.

SCG (0.2 g) was dissolved in 20 mL of purified water. Upon dissolution, the aqueous solution was mixed with a ready-to-use base (79.8 g) to obtain a 0.2% topical formulation. A similar protocol was used to prepare 1% topical formulations. In this case, 1 g of SCG was dissolved in 20 mL of purified water, followed by mixing in a mortar and pestle with 79 g of ready-to-use base. However, higher concentrations were not physically stable when using this protocol, and phase separation was observed immediately after preparation.

To overcome this issue, the water was partially replaced with propylene glycol as the wetting agent. SCG (2 g) was dispersed in propylene glycol (2 g), followed by mixing with the ready-to-use Creamgel base (96 g). In the case of Beeler’s base, SCG (2 g) was also dispersed in propylene glycol (2 g) and then mixed in a mortar and pestle with purified water (21 g) and ready-to-use Beeler’s base (75 g). The addition of water was required when using Beeler’s base to diminish the final viscosity of the formulation. Topical creams were manually stirred in a mortar and pestle until a homogenous formulation was visually formed. Afterward, the formulations were packaged in a 5 mL propylene plastic container and sent for analysis.

#### 2.2.2. Physicochemical Stability Studies

SCG formulations were stored at different temperatures and the samples were withdrawn at different times for long-term stability (15, 30, 60, 120, 180, 240, and 365 days). The topical formulations (TLCG and TLE) were stored at room temperature (25 ± 2 °C) and accelerated conditions (40 ± 2 °C) within stability chambers (Cuspor Ltd., Dublin, Ireland). These temperatures were selected based on the ICH guidelines for climatic zone Type II, to which Spain belongs [17]. The relative humidity was not taken into consideration for this study. The rest of the topical formulations (TM1CG, TM1BB, TM2CG, TM2BB) were kept at 25 °C and 40 °C, analyzed after 3 and 6 months. The chemical stability was quantified as the amount of SCG degraded over time and was fitted to a first-order degradation while the physical stability was expressed as visual appearance (color change).

#### 2.2.3. SCG Quantification by HPLC

The samples (0.5 g) were dispersed in 10 mL of mobile phase using a water bath ultrasound for 10 min. Subsequently, the samples were centrifuged (10 min at 3500× *g*) and the supernatant was filtered through a 0.45 µm Millipore PTFE filter. The filtrate was diluted with the mobile phase (1/50, *v*/*v*) and injected into the HPLC which was equipped with a Jasco PU-1580 pump, a Jasco AS-2050 Plus autosampler, and a Jasco UV-1575 UV-visible detector. The integration of the peaks was performed with the program Borwin 1.5 for PC (JMBS Developments). Chromatography was performed using a mixture of acetonitrile: 20 mM phosphate buffer pH 5 (25:75. *v*/*v*) as the mobile phase and a Hypersil BDS C18. 250 × 4.6 mm of 5 µm column. The elution of the samples was carried out using a flow of 1 mL/min and the detection was carried out in a UV-Vis detector module at 240 nm. The injection volume was 100 µL. The cromoglycate content was quantified by integrating the area under the curve. The retention time was 3.45 min. The analytical method was previously validated in-house. The linearity was good (R^2^ > 0.999) between 0.1 and 50 µg/mL (*y* = 331.4*x* + 32.5). The detection limit was 0.09 µg/mL, while the quantification limit was 0.3 µg/mL.

#### 2.2.4. Physicochemical Characterization of SCG Formulations

A particle size analyzer (Microtrac S3500, Microtrac, PA, USA) was used for the determination of the mean particle size and particle size distribution of SCG formulations. The size and distribution of the prepared formulations were expressed by the volume median diameter (MV) and the D_10_, D_50_, and D_90_ (indicating the percentages of particles having 10%, 50%, and 90% of the diameter equal to or lower than the given value). The span was calculated as a measure of polydispersity, using Equation (1) as follows:

Span = (D_90_ − D_10_)/D_50_ (1)

Before measurement, the SCG formulations were diluted (1/100) with deionized water. Each sample was measured in triplicate [18]. The rheological behavior of the formulations was evaluated in triplicate using a Brookfield (Middleborough, MA, USA) Model DV-III fitted with a temperature control probe. A 5 cm cone–plate measuring geometry was used. The temperature of all the measurements was maintained at 25 °C. The viscosity (cP) and shear stress (D × cm^−2^) were determined over a speed rate from 0 to 0.05 rpm, and a shear rate from 0 to 0.192 (Hz).

#### 2.2.5. In Vitro Permeation Studies Using Franz Cells

Topical formulations (TM) containing 1 and 2% SCG were tested. Strat-M^®^ membranes (Millipore, Billerica, MA, USA) were mounted between the donor and the receptor chamber of Franz diffusion cells (Soham Scientific, Soham, UK), with an effective diffusion area of 1.76 cm^2^ and a cell volume of 12 mL filled with freshly phosphate-buffered solution (PBS, pH 7.4). PBS was composed of 8 g NaCl, 0.2 g KCl, 1.19 g Na_2_HPO_4_, and 0.244 g KH_2_PO_4_ in 1 L at pH 7.4. The diffusion cells were maintained at 32 ± 0.5 °C, and the fluid in the receptor chambers was stirred continuously at 350 rpm. The SCG formulations were accurately weighed (500 mg) on an analytical Sartorius balance (0.1 mg resolution), and then loaded in the donor chambers and spread over a thin layer on the Strat-M^®^ membrane. The samples (1 mL) from the receptor chambers, at several time intervals (15 min, 30 min, 60 min, 90 min, 120 min, 180 min, 240 min, and 360 min) were withdrawn for HPLC determination without further dilution, and the volume was replaced immediately with a fresh phosphate buffer solution to keep sink conditions. The cumulative amounts of SCG that permeated through the Strat-M^®^ membrane were plotted as a function of time. Each formulation was tested in triplicate. Regression analysis was used to calculate the slopes and intercepts of the linear portion of each graph. The time required to reach a steady state was called the lag time (Lag). The lag time was determined by extrapolating the linear portion of the permeation *versus* the time curve of the time axis. The SCG flux through the membrane to the receptor compartment (µg/cm^2^/h) was calculated by diving the amount of the drug accumulated in the receptor compartment at each time by the diffusion area (*A*), which is the diameter of the Franz cell. Fick’s first law was derived to calculate flux (J) at a steady state [18,19,20] (Equation (2)):

(2) $$Jss=\frac{dQ}{dt}\frac{1}{A}$$

where *dQ*/*dt* is the rate of SCG permeation across the skin (µg/h)*,* and *A* the diffusion area (cm^2^).

#### 2.2.6. In Vitro Permeation Studies Using Porcine Skin

Porcine ear skin was removed from a male pig (6 months old) sacrificed at the local slaughtered house (Madrid, Spain). The removal of the skin from cartilage was performed according to previously published protocols [21]. The skin was mounted between the donor and receptor chamber of a Franz diffusion cell and equilibrated with PBS for 30 min. The experiments were performed in triplicate with TM32CG and TM2BB, and a similar methodology was utilized when the artificial skin membranes (Strat-M^®^ membrane, Millipore, Billerica, MA, USA) were used.

#### 2.2.7. Data Analysis

Data analysis (calculation of the mean values and standard deviations) was performed using Excel (Microsoft Office 2021). For the statistical analysis, Minitab 19 (Minitab Ltd., Coventry, UK) was used with a one-sided ANOVA, considering significant differences with a *p*-value less than 0.05. From the stability data, the degradation constants at different temperatures were calculated by adjusting to apparent first-order kinetics [21,22].

## 3. Results

### 3.1. Physicochemical Stability

**Figure 2:**
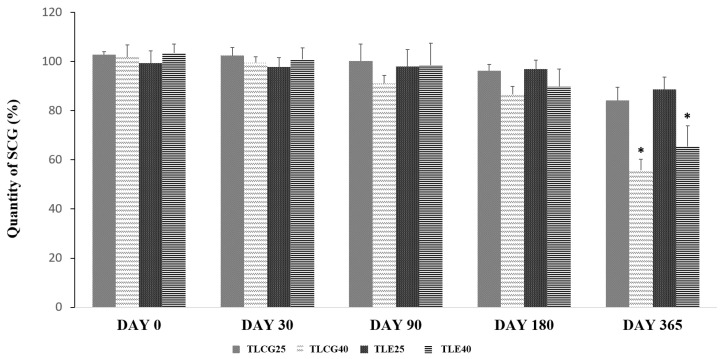
Chemical stability of topical SCG formulations at 0.2% over one year. Key: TLCG25: Creamgel stored at 25 °C; TLCG40: Creamgel stored at 40 °C; TLE25: Eucerinum^®^-based cream stored at 25 °C; TLE40: Eucerinum^®^-based cream stored at 40 °C. * Statistical significance: *p* < 0.05.

No significant degradation of SCG was observed (≤90%) at 25 °C (storage condition A) nor under forced conditions at 40 °C (storage condition B) for up to 3 months in the creams containing 0.2% SCG. After 6 months, a significant degradation (*p*-value < 0.05) above 10% was observed in those formulations stored at 40 °C (Figure 2). The formulations underwent a pronounced color change after 12-month storage at 40 °C. No statistically significant differences were observed between the Creamgel and Eucerinum^®^ formulations.

The degradation kinetics of SCG was fitted to a first-order reaction. The degradation rate increased between three to four folds when the formulations were stored at a higher temperature (40 °C). In the Creamgel formulation, the SCG degradation rate was 0.018%/month at 25 °C and 0.057%/month at 40 °C, while in the Eucerinum cream, the SCG degradation rate was 0.012 and 0.045%/month at 25 and 40 °C, respectively.

All TM formulations prepared at 1 or 2% using Creamgel or Beeler’s base as vehicles exhibited a similar stability to 0.2% SCG creams when stored at 25 °C for 6 months (drug content > 90%).

### 3.2. Permeability Assays

**Figure 3:**
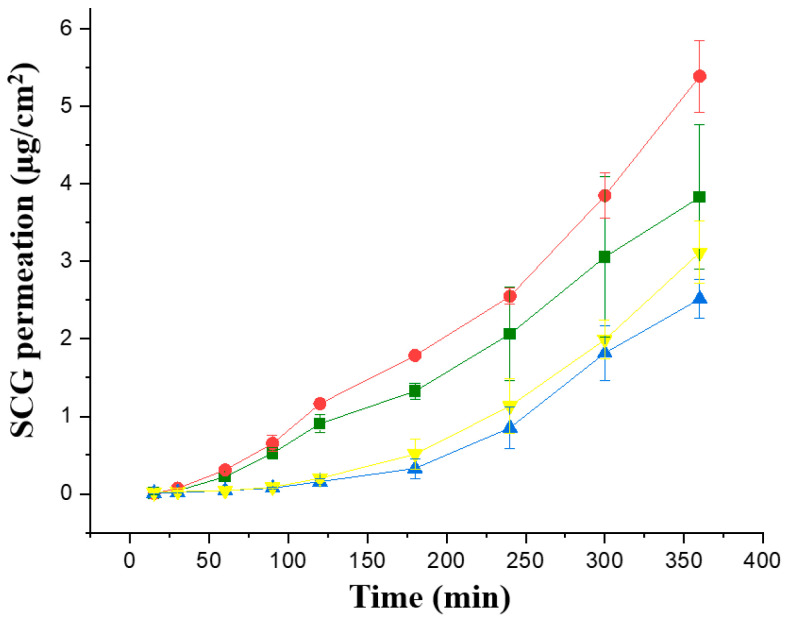
In vitro permeability of SCG formulations across Strat-M^®^ membrane; Key: Creamgel SCG 1% (-■-) (TM1CG), Beeler’s base SCG 1% (-▲-) (TM1BB), Creamgel SCG 2% cream (-●-) (TM2CG), and Beeler’s base SCG 2% (-▼-) (TM2BB).

The first Franz cell study was performed in the four topical formulations with a greater concentration of SCG, 1 and 2%, to enhance the topical SCG bioavailability. Permeability studies were performed initially across in vitro commercially available membranes, Strat-membranes, that closely mimic the human skin. When comparing the J_ss_ steady flux, the formulations prepared with Creamgel showed a significantly higher permeability across synthetic membranes (1.58 and 1.77-fold greater) in contrast to Beeler’s base (Figure 3, Table 2). In addition, there was a significantly larger permeability when the SCG concentration was doubled. In this case, the permeability of Creamgel and Beeler’s base formulations at 2% showed a 1.34- and 1.2-fold higher permeability (in terms of J_ss_ flux) than the 1% SCG formulations, respectively.

**Figure 4:**
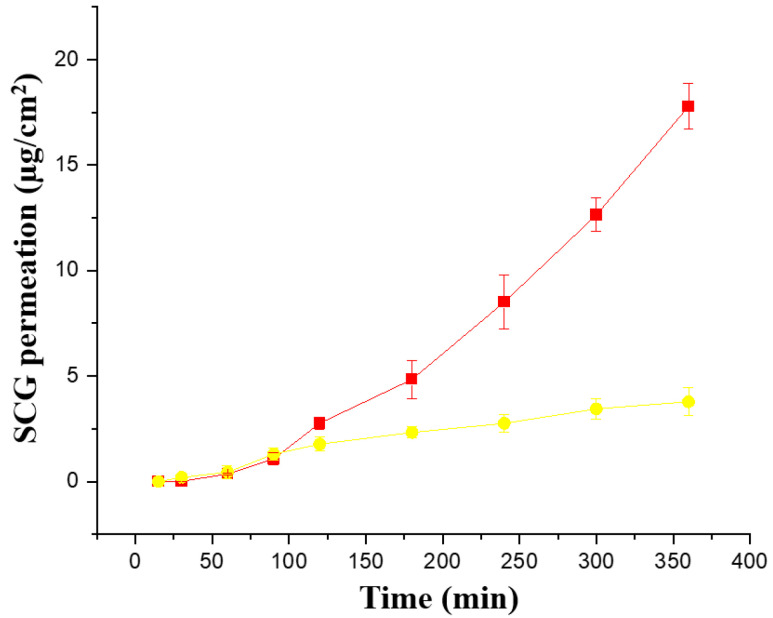
Ex vivo permeability of SCG formulations across pig ear skin. Key: Creamgel SCG 2% cream (-■-) (TM2CG), and Beeler’s base SCG 2% (-●-) (TM2BB).

Ex vivo permeability across pig ear skin was performed with both formulations (Creamgel and Beeler’s base) with 2% SCG (Figure 4). Interestingly, both formulations showed a better performance compared to synthetic membranes. The Creamgel formulation with 2% SCG showed a 3.4-fold higher permeability (expressed as J_ss_ flux) across pig’s skin, while Beerler’s base formulation at 2% of SCG showed a 1.3-fold enhancement compared to the permeability across synthetic Strat-membranes^®^ at the same concentration. The lag time was superior with Beeler’s base formulations (approx. one hour). Creamgel formulations also required prolonged periods (>30 min) to show permeability across the skin.

### 3.3. Physicochemical Properties: Particle Size and Viscosity

**Figure 5:**
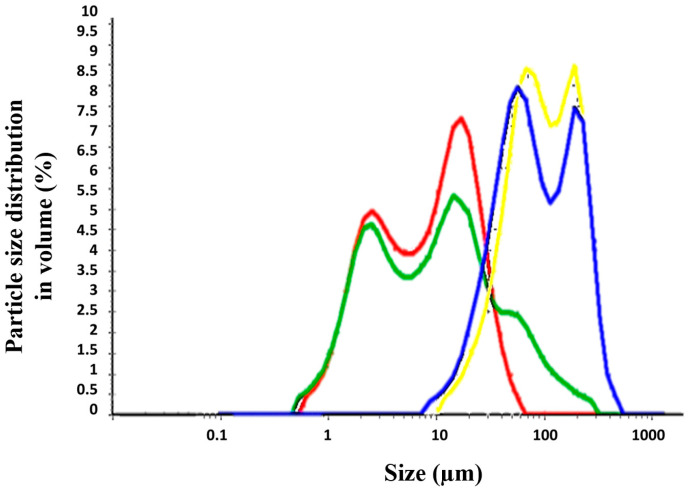
Particle size distribution of SCG topical formulations. Key: Creamgel SCG 1% (―) (TM1CG), Beeler’s base SCG 1% (―) (TM1BB), Creamgel SCG 2% cream (―) (TM2CG), and Beeler’s base SCG 2% (―) (TM2BB).

The different permeability across Strat-M^®^ membranes and pig’s skin can be explained based on the different physicochemical properties of the formulations, such as particle size and viscosity. Topical formulations (TM) were dispersed in water and the particle size distribution was evaluated (Table 3 and Figure 5). The Creamgel formulations exhibited a median particle size significantly smaller (8–10-fold lower) than those formulations containing Beeler’s base. However, both types of formulations were bimodal, exhibiting two different particle size populations. Creamgel formulations exhibited a small particle size fraction of 2 µm, along with a 15 µm population, while Beeler’s base formulations exhibited significantly greater populations of 50 and 170 µm, respectively, with an overall greater polydispersity. Possibly, the appearance of a second particle size population corresponds with SCG-loaded droplets.

**Figure 6:**
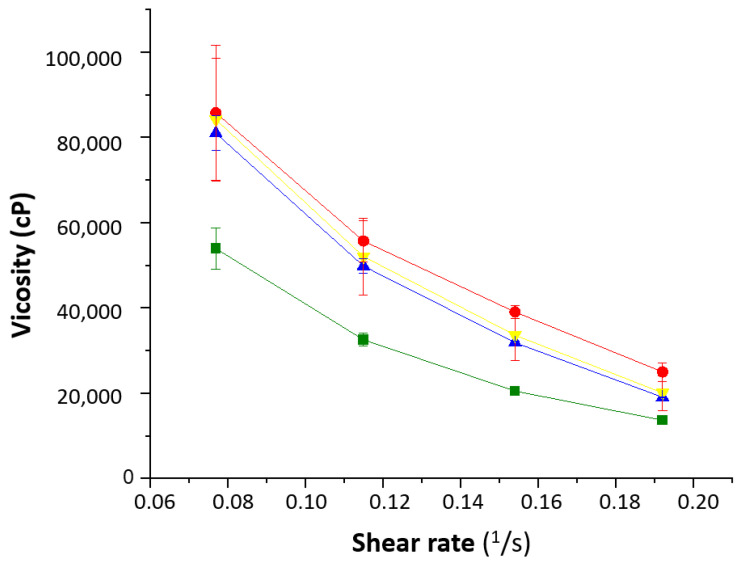
Rheological behavior of the SCG formulations. Key: Creamgel SCG 1% (-■-) (TM1CG), Beeler’s base SCG 1% (-▲-) (TM1BB), Creamgel SCG 2% cream (-●-) (TM2CG), and Beeler’s base SCG 2% (-▼-) (TM2BB).

The rheological behavior of the formulations showed that the water content played an important role (Figure 6). The 1% Creamgel formulation showed a lower viscosity even at lower shear rates. However, a significant increase in viscosity was observed between the 1 and 2% Creamgel formulations. This effect can be related to the replacement of water with the Creamgel base. However, the rheological profile of Beeler’s base formulations was kept constant, which can be attributed to the fact that water was also used to formulate 2% Beeler’s base formulations.

## 4. Discussion

Mast cell activation is a common and necessary process for the maintenance of survival. However, when mast cells are pathologically overproduced or activated, it results in certain disorders that can range from very rare to very common. On the common side, allergic rhinitis and asthma can affect up to 10 to 30% of the population, while mastocytosis and mast cell activation syndrome [23] are considered rare diseases according to the European Union, which means that less than 1 person is affected in 2000 people [24]. Oral SCG is used in patients with mastocytosis with gastrointestinal symptoms, as it has been shown to effectively reduce these symptoms [24,25,26,27], whereas topical treatments can reduce pruritus and the flaring of skin lesions.

Access to commercially available SCG treatments differs from country to country [28]. In Spain, there are no industrially fabricated treatments with SCG for topical administration and patients rely on the preparation of extemporaneous compounding formulations in hospital and community pharmacies. The shelf life has not been determined in most of the formulations prepared in clinical settings and hence, patients need to regularly collect their medicines from the points of care. This results in an unnecessary waste of medicine and can put patient compliance at risk. In this work, for the first time, the stability of several commonly prepared extemporaneous compounding formulations for topical administration has been determined. All the tested topical formulations were shown to be stable for at least 6 months and contain preservatives to prevent microbiological contamination. The microbiological growth is not expected to be an issue in any of the formulations assessed, as the ready-to-use bases contain preservatives. However, according to the Pharmaceutical Professional Consortium in Granada (Spain), it is recommended not to exceed a three-month shelf-life at 25 °C for emulsions packaged in an open jar container. Stability can be increased up to 6 months if packing is performed in a tube that avoids external contamination [29].

Another key element demonstrated in this work is the importance of choosing a suitable vehicle for the preparation of topical formulations. Other authors have tested different topical formulations with different vehicles across artificial membranes of cellulose esters, obtaining greater flux permeability rates at greater concentrations [30]. In Spain, dermatologists tend to prescribe topical formulations containing 0.2% SCG. Those formulations are efficacious in infants and children, but with a reduced effect on adults suffering from mast cell diseases with skin symptoms. In other countries, topical formulations up to 4% SCG have been formulated, showing a significant reduction in erythema and pruritus, but not in the weal volume or temperature increase [2]. This effect in reducing pruritus supports the hypothesis that SCG acts by inhibiting the sensory C-fiber nerve activation of the skin rather than preventing mast cell degranulation. The lack of efficacy on weals is attributed to the poor capacity of the vehicle utilized in the preparation of the topical formulations unable to penetrate across the stratum corneum and reach the mast cell accumulated in the outermost layer and interface of the epidermis [12].

Creamgel base was selected in the first stability study over an Eucerinum base. Both bases contain dimethicone which is a gentle, effective moisturizing ingredient that provides a long-lasting skin-smoothing effect. Unlike Eucerinum, Creamgel has butylene glycol that acts as a humectant and emollient by creating a skin barrier, preventing water loss. Additionally, butylene glycol improves penetration. Based on our clinical experience, patients using 0.2% topical formulations preferred the Creamgel over the Eucerinum formulation. The Creamgel formulation was perceived as a cream that was easier to apply and spread on the skin, necessitating less force. Patients suffering from mastocytosis have painful lesions on the skin and hence, the feeling during the administration of topical formulations is essential to ensure patient compliance. Eucerinum^®^ base was replaced with Beeler’s base, which is a common base utilized for the preparation of multiple extemporaneous topical formulations in community pharmacies in Spain. Creamgel has elicited better SCG skin permeation compared to conventional Beeler’s base, which is due to the different components of both vehicles. This effect can be related to the combination of cetyl alcohol with polysorbate [31], linked to the smaller droplet size formed upon dilution in water and the lower viscosity of the formulations. Surprisingly, the permeability of Creamgel formulations at 2% across the pig skin was significantly higher (3-fold increase) than across synthetic membranes. Previously, salcaprozate sodium has been used as a permeation enhancer for the topical delivery of SCG [11]. Compared to salcaprozate sodium, Creamgel has exhibited a much greater permeation rate across pig skin, with a lower lag time. There is a clear concentration gradient when using Creamgel vehicle, which can be useful when prescribing a topical formulation in patients with different characteristics and symptomatology. It is well known that infants have a more permeable stratum corneum and hence, a cream with a lower concentration may be sufficient to elicit a pharmacological effect, while in adults, higher concentrations may be required. Nevertheless, clinical studies should be performed to evaluate the pharmacological profile of Creamgel formulations prepared at higher concentrations than the conventional creams at 0.2% SCG.

## 5. Conclusions

The stability of topical extemporaneous compounded SCG formulations can be extended for up to three months at room temperature (25 °C). The selection of a suitable vehicle for the preparation of topical formulations is key as it determines the permeability across the skin. Creamgel, a commercially available vehicle, has been shown to significantly improve topical permeability compared to Beeler’s base cream. At higher SCG concentrations, the permeability across both synthetic and pig skin is enhanced significantly. This would allow dermatologists and other specialists to make prescriptions depending on the patient’s characteristics and the need for SCG delivered across the stratum corneum.

## Figures and Tables

**Table 1:** Composition of topical SCG formulations.

Formulation ID	SCG (%)	Propylene Glycol	Eucrinum^®^	Acofar Creamgel	Beeler’s Base
TLCG	0.2	-	-	Yes	-
TLE	0.2	-	Yes	-	-
TM1CG	1	-	-	Yes	-
TM1BB	1	-	-	-	Yes
TM2CG	2	Yes	-	Yes	-
TM2BB	2	Yes	-	-	Yes

**Table 2:** Comparison of skin permeability parameters of SCG formulations across synthetic Strat-M membrane^®^ (S.M.) and pig ear skin (P.S.) Key: CG, Creamgel, BB, Beeler’s base.

Formulation	Membrane	J_ss_ (µg/cm^2^/h)	Lag Time (h)
TM1CG	S.M.	0.666 ± 0.123	0.6 ± 0.1
TM1BB	S.M.	0.420 ± 0.104	1.1 ± 0.2
TM2CG	S.M.	0.894 ± 0.25	0.6 ± 0.2
TM2BB	S.M.	0.504 ± 0.113	1.1 ± 0.2
TM2CG	P.S.	3.042 ± 0.487	0.8 ± 0.2
TM2BB	P.S.	0.672 ± 0.264	1.2 ± 0.5

**Table 3:** Particle size and viscosity parameters. Mean particle size is expressed in volume (mean ± SD). The volume distribution (MV) is the distribution per volume of the particle size, shown as a differential of the total volume of all counts. * Viscosity value is reported at 0.05 rpm.

	MV						Population 1				Population 2				SPAN	Viscosity (cP) *
Formulation	D_10_		D_50_		D_90_		Diameter (µm)		Volume (%)		Diameter (µm)		Volume (%)			
	Mean	SD	Mean	SD	Mean	SD	Mean	SD	Mean	SD	Mean	SD	Mean	SD		
TM1CG	1.7	0.0	8.8	0.3	27.2	10.6	14.2	0.3	68.8	0.7	2.2	0.0	31.2	0.7	2.89	13,631
TM1BB	35.3	0.5	96.0	2.2	235.5	3.0	177.9	1.1	46.2	1.0	58.6	0.4	53.8	1.0	4.89	19,005
TM2CG	1.7	0.0	10.1	0.3	51.1	9.8	16.5	0.2	69.4	0.6	2.1	0.0	30.6	0.6	2.08	24,910
TM2BB	23.0	2.8	80.5	2.2	231.0	4.8	165.9	5.9	50.7	2.2	39.9	3.6	49.2	2.2	2.58	20,030

## Data Availability

Not applicable.
